# Hypoxia-induced CREB cooperates MMSET to modify chromatin and promote DKK1 expression in multiple myeloma

**DOI:** 10.1038/s41388-020-01590-8

**Published:** 2021-01-08

**Authors:** Yinyin Xu, Jing Guo, Jing Liu, Ying Xie, Xin Li, Hongmei Jiang, Jingjing Wang, Ziyi Peng, Jingya Wang, Sheng Wang, Chao Wan, Lanting Chen, Yuping Zhong, Beizhong Liu, Zhiqiang Liu

**Affiliations:** 1 Clinical Laboratory of Yongchuan Hospital, Chongqing Medical University, Chongqing, China; 2 Key Laboratory of Laboratory Medical Diagnostics, Ministry of Education, Department of Laboratory Medicine, Chongqing Medical University, Chongqing, China; 3 The Province and Ministry Co-sponsored Collaborative Innovation Center for Medical Epigenetics, Tianjin Key Laboratory of Cellular Homeostasis and Human Diseases, Department of Physiology and Pathophysiology, School of Basic Medical Science, Tianjin Medical University, Heping, Tianjin, China; 4 Department of Hematology, Yongchuan Hospital of Chongqing Medical University, Chongqing, China; 5 Department of Hematology, Qingdao Municipal Hospital, Qingdao, Shandong China; 6 Tianjin Medical University Cancer Institute and Hospital, National Clinical Research Center for Cancer, Tianjin Key Laboratory of Cancer Prevention and Therapy, Tianjin’s Clinical Research Center for Cancer, Tianjin, China

**Keywords:** Methylation, Prognostic markers

## Abstract

Myeloma cells produce excessive levels of dickkopf-1 (DKK1), which mediates the inhibition of Wnt signaling in osteoblasts, leading to multiple myeloma (MM) bone disease. Nevertheless, the precise mechanisms underlying DKK1 overexpression in myeloma remain incompletely understood. Herein, we provide evidence that hypoxia promotes *DKK1* expression in myeloma cells. Under hypoxic conditions, p38 kinase phosphorylated cAMP-responsive element-binding protein (CREB) and drove its nuclear import to activate *DKK1* transcription. In addition, high levels of DKK1 were associated with the presence of focal bone lesions in patients with t(4;14) MM, overexpressing the histone methyltransferase MMSET, which was identified as a downstream target gene of hypoxia-inducible factor (HIF)-1α. Furthermore, we found that CREB could recruit MMSET, leading to the stabilization of HIF-1α protein and the increased dimethylation of histone H3 at lysine 36 on the DKK1 promoter. Knockdown of CREB in myeloma cells alleviated the suppression of osteoblastogenesis by myeloma-secreted DKK1 in vitro. Combined treatment with a CREB inhibitor and the hypoxia-activated prodrug TH-302 (evofosfamide) significantly reduced MM-induced bone destruction in vivo. Taken together, our findings reveal that hypoxia and a cytogenetic abnormality regulate DKK1 expression in myeloma cells, and provide an additional rationale for the development of therapeutic strategies that interrupt DKK1 to cure MM.

## Background

Myeloma bone disease is the most frequent feature of myeloma, occurring in approximately two-thirds of patients at diagnosis and in nearly 90% of patients with multiple myeloma (MM) [1]. Investigations have revealed that the pathogenesis of bone disease in MM is caused by enhanced osteoclastic activity and weakened osteoblastic bone formation [2]. The functional exhaustion of osteoblasts is found in active myeloma, and myeloma cells can block the differentiation of osteoblasts and induce prompt apoptosis [3]. Myeloma bone disease severely impairs patients’ quality of life and is a major cause of disability and mortality. It is reasonable to expect that alleviating bone loss should improve the quality of life and survival of patients with MM [4].

The complicated mechanism of myeloma bone disease makes therapy very challenging. To date, several cytokines have been identified for the inhibition of osteoblastogenesis and activity in myeloma bone disease, including dickkopf-1 (DKK1), monocyte chemotactic protein-1, interleukin (IL)-3, receptor activator of NF-κB ligand, tumor necrosis factor α, soluble frizzled-related protein-3, and sclerostin [4, 5]. The secreted DKK family members are naturally occurring antagonists of Wnt/β-catenin signaling and comprise four members in vertebrates (DKK1–4). DKK1 prevents the activation of Wnt signaling by binding to the Wnt co-receptor LRP5/6, masking the active sites of LRP5/6 and triggering their endocytosis, thereby making it unavailable for interactions with Wnt ligands [6]. Over the past decade, DKK1 has emerged as an important factor in multiple critical aspects of bone biology. Several animal experiments and preclinical trials have revealed that DKK1 can repress osteoblasts and stimulate osteoclasts, causing an imbalance in bone metabolism [7, 8]. Transgenic mice overexpressing *Dkk1* develop osteopenia, whereas mice haploinsufficient for *Dkk1* exhibit high bone mass [9]. Novel therapeutic neutralizing antibodies have been tested as bone anabolic agents; however, their effects on the activation of the Wnt/β-catenin pathway to rehabilitate bone disruption remain to be fully clarified [10].

DKK1 is frequently found to be overexpressed in the myeloma microenvironment of patients with serious bone disease, but it is highly restricted in normal tissues [11]. A previous study presented evidence that DKK1 expression in MM plasma cells is, in part, dependent on JNK signaling, and the oxidative stress response regulates DKK1 expression through the JNK signaling cascade [12]. Marrow plasma from patients with myeloma with >12 ng/ml of DKK1 interrupts the osteoblastic differentiation of murine mesenchymal stem cells (MSCs) in vitro [7]. A neutralizing antibody against DKK1 prevents the development of osteolytic bone disease in MM and promotes bone fracture healing, and it simultaneously inhibits myeloma growth in vivo [13–16]. DKK1 has a clear and vital role in the pathogenesis of myeloma bone disease. Intriguingly, Tosi et al. reported that bone resorption was more prominent in patients with t(4;14) chromosomal abnormality [17], a karyotypically silent molecular abnormality that has been described in 10–20% of newly diagnosed MM and drives overexpression of *MMSET* and *FGFR3* [18]. This suggests a possible correlation between these abnormally expressed genes and myeloma-related bone disease, but how they regulate DKK1 expression is unknown. Therefore, understanding of the underlying mechanism for universal DKK1 expression in MM may provide tools for preventing this disease and the development of osteolytic bone lesions in myeloma.

## Results

### Gene expression profile analysis revealed elevated DKK1 expression in myeloma cells in response to hypoxia

**Fig. 1:**
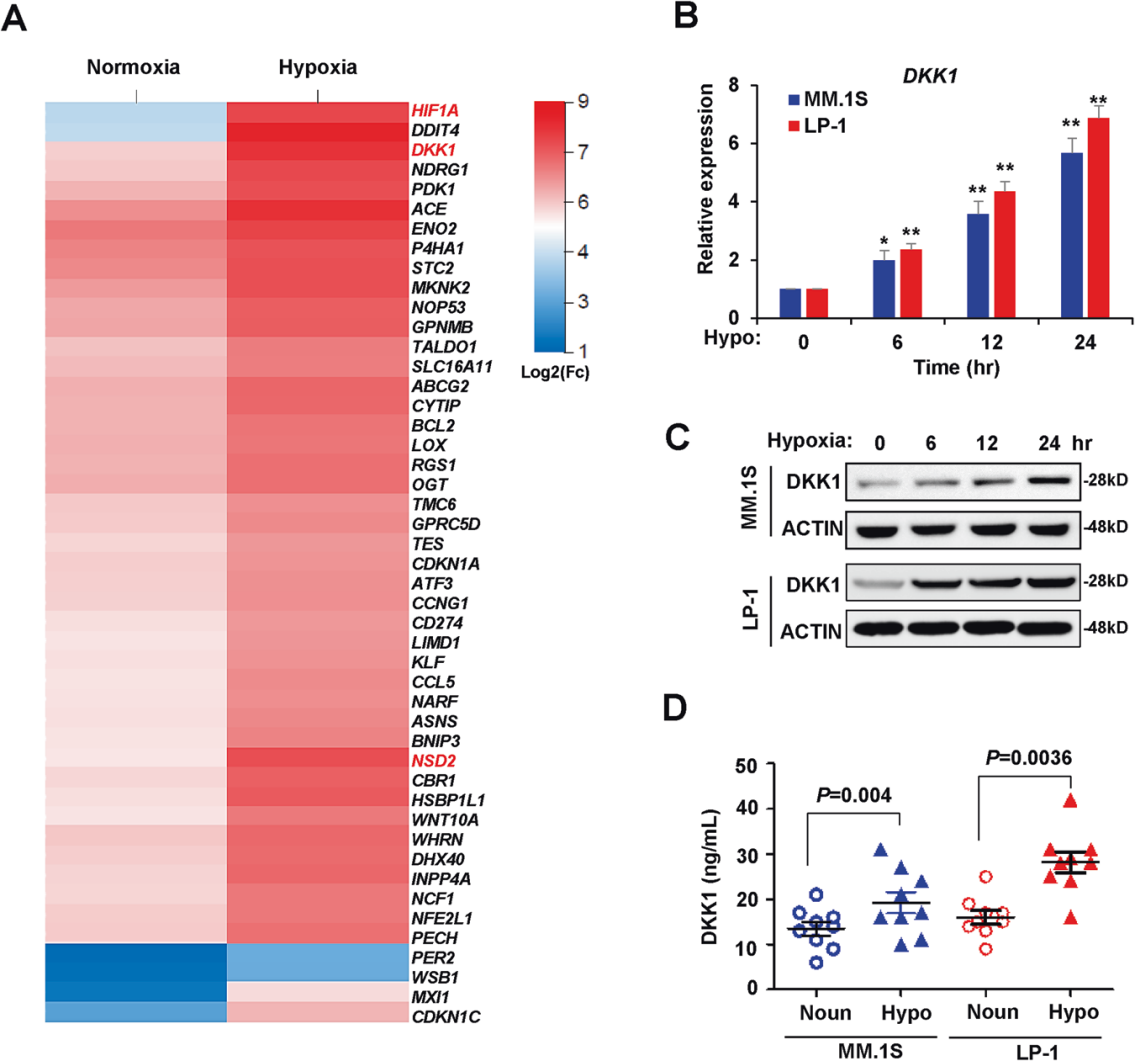
*DKK1* expression is augmented in hypoxia-treated myeloma cells. **a** Heat map shows the top highly expressed genes in overlap of MM.1S and LP-1 cells under stimulation of hypoxia for 24 h (*n* = 3 for each cells; color scale, centered and scaled log2(FC) values). **b** *DKK1* mRNA expression by qPCR and **c** DKK1 protein level by Western blot in MM.1S and LP-1 cells under conditions of hypoxia (1% O_2_) for 6, 12, and 24 h (qPCR, *n* = 3 with each detection triplicated; WB, representative image for at least three independent experiments). **P < 0.01 determined by one-way ANOVA. **d** ELISA assay shows the secreted DKK1 level in culture medium of MM cells under normoxia and hypoxia conditions. *n* = 3 with each detection triplicated*, P* values were determined by Student’s *t-*test.

Recent studies have revealed the important roles of hypoxia in promoting myeloma drug resistance and metastasis from bone marrow into peripheral blood [19, 20], but gene expression profiles have not been investigated. We cultured MM.1S and LP-1 cells in a hypoxic chamber and profiled gene expression using bulk cell RNA sequencing. We assessed differential gene expression with the significance criterion of an adjusted *P* value < 0.01 and abs (log 2FC) > 1. We identified 1536 differently expressed genes in MM.1S cells and 987 genes in LP-1 cells, and 47 significantly increased genes among 58 genes overlapped between these two cell lines (Supplementary Fig. 1A; and Supplementary Table 2). Among them, *DKK1*, ATP-binding cassette subfamily G member 2 (junior blood group) (*ABCG2*), nuclear receptor-binding SET domain protein 2 (*NSD2*), and BCL2 apoptosis regulator (*BCL2*), which are closely related to myeloma progression and bone lesions, were included in the highly elevated genes, and activating transcription factor 3 (*ATF3*), MAX interactor 1, dimerization protein (*MXI1*), and stanniocalcin 2 (*STC2*) are known targets of the hypoxia-inducible factor (HIF)-1α signaling pathway (Fig. 1a). The mostly affected pathways under hypoxia according to KEGG analysis suggested a correlation between hypoxia and mitogen-activated protein kinase (MAPK) signaling (Supplementary Fig. 1b). We confirmed that hypoxia induced the expression of *DKK1* at the mRNA and protein levels in MM cell lines with different cytogenetic backgrounds (Fig. 1b, c; and Supplementary Fig. 1C). Enzyme-linked immunosorbent assay (ELISA) also confirmed that hypoxia promoted the secretion of DKK1 into culture medium (Fig. 1d).

### The hypoxia-MAPK-p38-CREB axis regulates DKK1 expression in myeloma cells

**Fig. 2:**
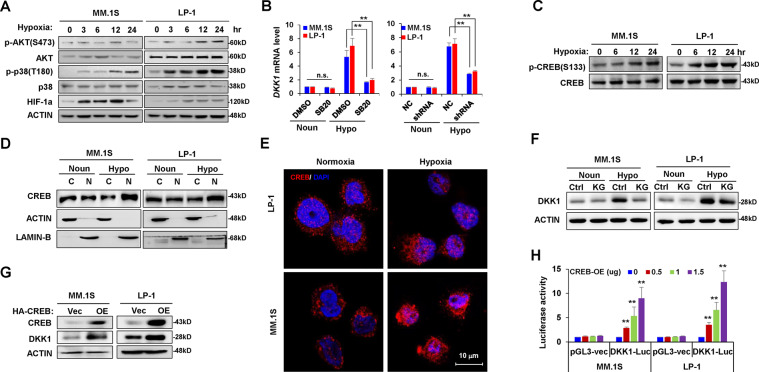
Hypoxia regulates *DKK1* expression via p38-CREB axis. **a** Western blot shows phosphorylation of AKT and p38 kinases in MM.1S and LP-1 cells incubated in a hypoxic condition for up to 24 h. **b** *DKK1* mRNA expression in MM cells incubated with 0.5 μM SB203580 for 24 h (left), or knockdown of p38a (shRNA #1) under normoxia and hypoxia conditions. n.s., no significance; **P < 0.001 by Student’s *t*-test for *n* = 3 independent experiments with each detection triplicated. **c** Western blot shows phosphorylation of CREB at Ser133 in MM cells exposed to normoxia or hypoxia for up to 24 h. **d** Cyto-nuclear translocalization of CREB under normoxia (Noun) and hypoxia (Hypo) conditions. Actin was used as the cytoplasm control and Lamin B was used as the nuclear control. **e** Subcellular localization of CREB under normoxia and hypoxia conditions by confocal immunofluorescence assay. **f** DKK1 level in MM cells under normoxia (Noun) or hypoxia condition (Hypo) treated with 10 μM of CREB inhibitor KG-501 (KG) or DMSO (Ctrl) for 24 h. **g** Western blot shows the efficacy of CREB overexpression and DKK1 expression in MM cells using lentivirus infection after 72 h. Representative Western blot images are for at least three independent experiments. **h** Luciferase assay shows the activity of pGL3-DKK1-luc reporter in the presence of increasing CREB overexpression (CREB-OE). pGL3-basic vector was used as control. **P < 0.001 by one-way ANOVA for three independent experiments.

A previous study showed that the p38 and AKT kinases are responders to hypoxia [21]. Herein, our results indicated that p38, but not AKT kinase, was most obviously activated after exposure of myeloma cells to hypoxia (Fig. 2a). Using a specific inhibitor of the p38 cascade, SB203580, or knocking down the p38 expression using short hairpin RNA (shRNA) (Supplementary Fig. 2A), reversed *DKK1* induction under hypoxia (Fig. 2b). Given that CREB is an important modulator of the p38 pathway, we investigated CREB activation under hypoxic conditions. Exposure to hypoxia gradually enhanced CREB phosphorylation at Ser133 in diverse MM cell lines (Fig. 2c; and Supplementary Fig. 2B) and accelerated the cyto-nuclear translocation of CREB as detected by analysis of nuclear and cytoplasmic proteins (Fig. 2d) and immunofluorescence staining (Fig. 2e). KG501, a CREB inhibitor, reversed DKK1 expression in myeloma cells exposed to hypoxia (Fig. 2f), and CREB overexpression upregulated DKK1 in myeloma cells (Fig. 2g). As CREB binds to palindromic (TGACGTCA) or half-site (TGAC/G) cyclic AMP response elements to regulate gene expression [22], we identified a typical CREB site (−1270) located in the proximal promoter of *DKK1* and then constructed a luciferase reporter driven by the *DKK1* promoter. CREB overexpression stimulated the transcriptional activity of the *DKK1*-luciferase reporter (Fig. 2h).

### Hypoxia induces HIF-1α to regulate MMSET expression in myeloma cells

**Fig. 3:**
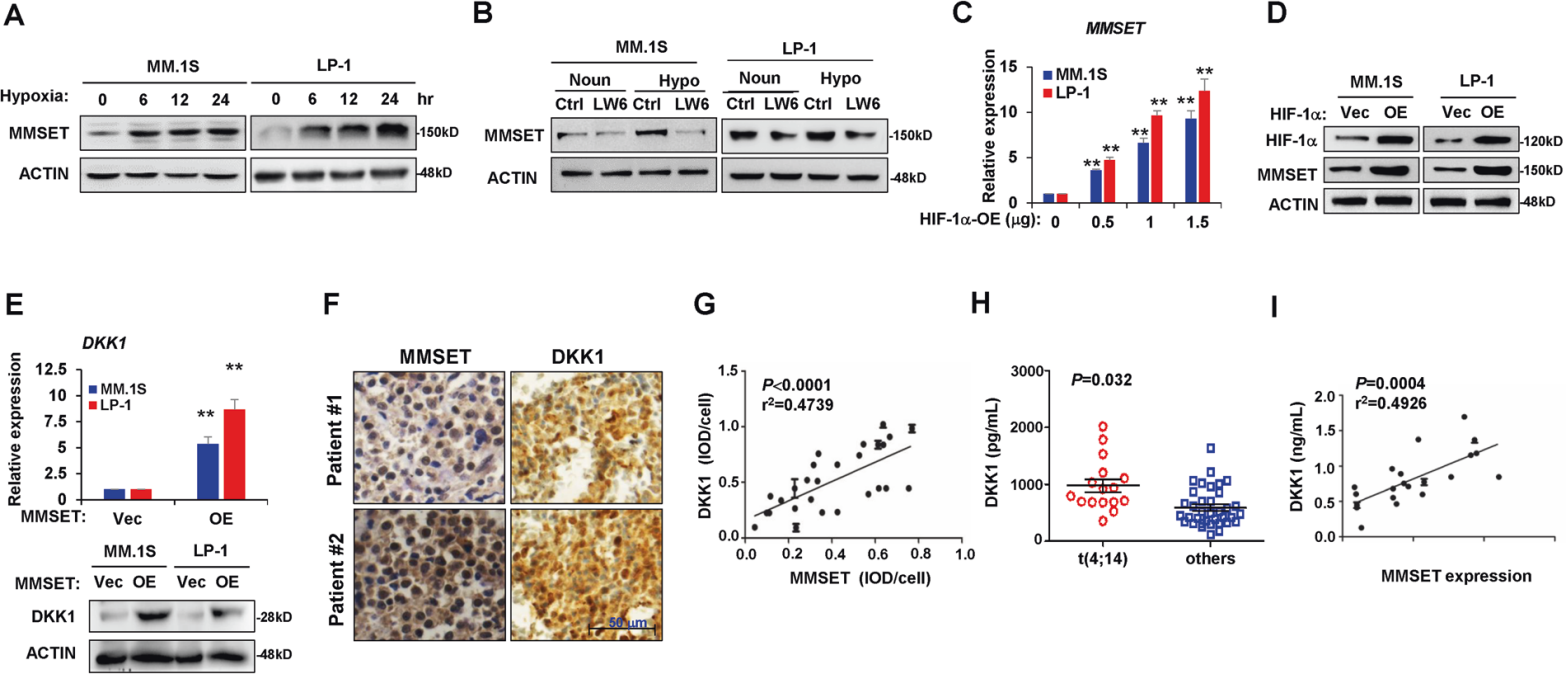
HIF-1α induces MMSET expression in MM cells. Western blot shows **a** MMSET protein levels in MM cells in hypoxia condition for up to 24 h and **b** MMSET level in MM cells treated with 10 μM of HIF-1α inhibitor LW6 for 24 h. *MMSET* mRNA expression by qPCR (**c**) and protein level by Western blot (**d**) in MM cells infected with lentivirus carrying HIF-1α overexpressing (OE) for 72 h. **P < 0.001 by one-way ANOVA for *n* = 3 independent experiments. (**e**) DKK1 expression in MM cells after lentivirus-mediated MMSET overexpression for 72 h by qPCR (upper penal) and by Western blot (lower panel). **P < 0.001 by Student’s *t*-test for *n* = 3 independent experiments. **f** Representative immunohistochemical staining for MMSET and DKK1 proteins in bone marrow biopsy specimens of the same patients. Scale bar, 50 μm. **g** Correlation coefficient between DKK1 and MMSET protein level in specimen of patients with MM (*n* = 26). *P* values were determined by Pearson coefficient. **P < 0.001 by one-way ANOVA for at least three independent experiments. **h** ELISA assay shows DKK1 level in bone marrow plasma of patients with t(4; 14) and other cytogenetic abnormal MM. **i** Correlation coefficient between bone marrow DKK1 level and MMSET expression in plasma cells of patients with MM with t(4; 14) (*n* = 17). *P* values were determined by Pearson coefficient.

MMSET is an important histone methyltransferase that facilitates the oncogenesis and malignancy of myeloma. Herein, our results showed that MMSET protein levels could be induced gradually by hypoxia compared with normoxia (Fig. 3a). In addition, we found that LW6, a HIF-1α inhibitor, inhibited the induction of MMSET in response to hypoxia (Fig. 3b). The forced expression of HIF-1α directly upregulated MMSET expression in myeloma cells at the mRNA and protein levels (Fig. 3c, d). Subsequently, we investigated the effect of MMSET on *DKK1* expression. Lentivirus-mediated MMSET overexpression enhanced *DKK1* expression at the mRNA and protein levels (Fig. 3e). Consistently, immunohistochemical analysis of bone marrow biopsy samples also indicated an association between MMSET abundance and DKK1 level in the same specimen (Fig. 3f). Taken together, our results demonstrated that hypoxia induces HIF-1α to promote MMSET expression in myeloma cells, ultimately contributing to the upregulation of DKK1.

The t(4;14) cytogenetic abnormality in patients with myeloma usually results in excessive MMSET expression. We found a strong positive correlation between DKK1 and MMSET expression in myeloma cells (Fig. 3g), and we observed that DKK1 levels were significantly higher in bone marrow plasma from t(4;14)-positive patients compared with patients with other chromatin abnormalities (Fig. 3h). Importantly, there was a strong positive correlation between DKK1 expression in myeloma cells and MMSET levels in patients with t(4;14) MM (Fig. 3i).

### MMSET stabilizes CREB

**Fig. 4:**
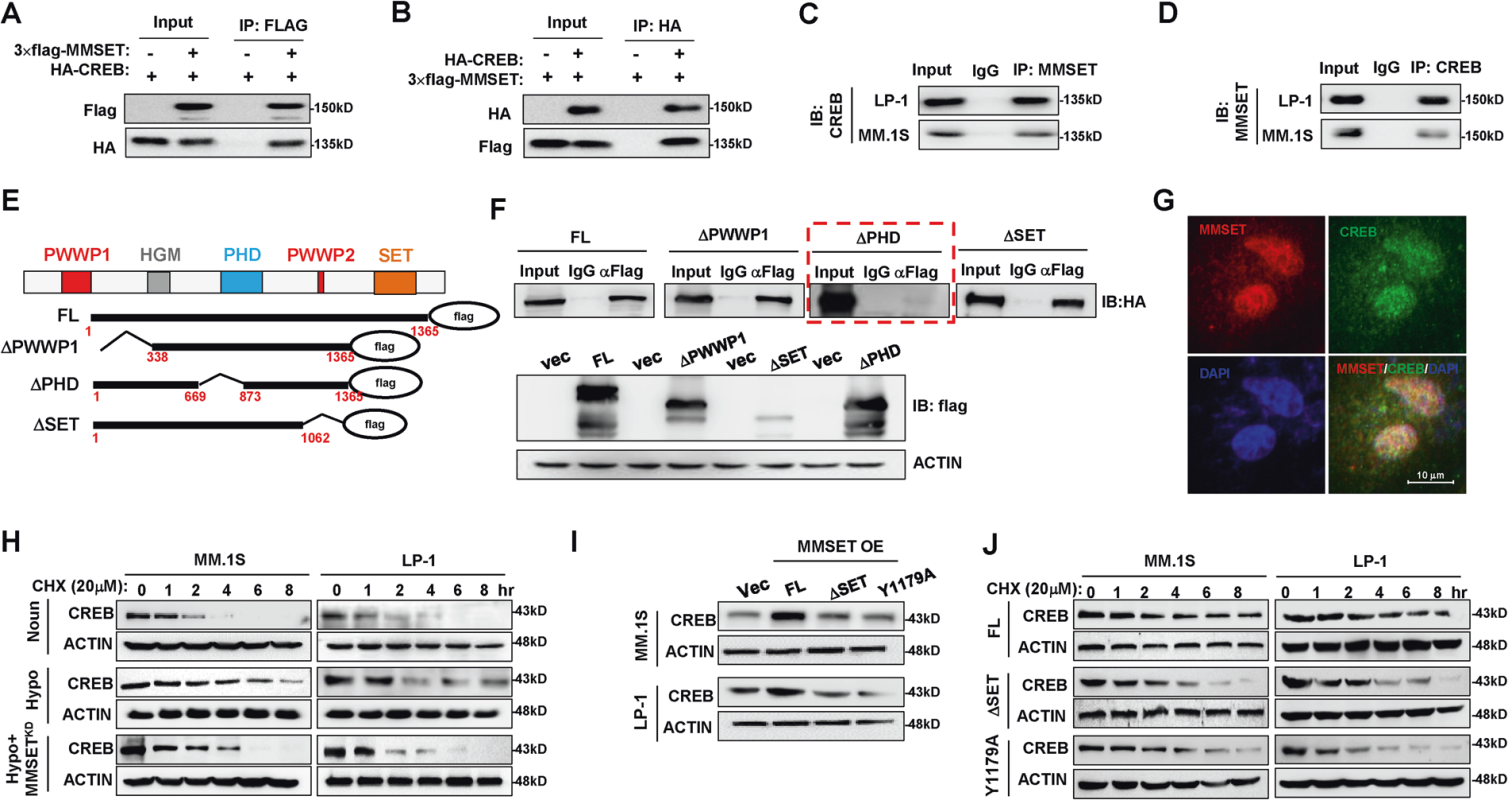
CREB physically interacts with MMSET in myeloma. **a** Interaction between exogenous FLAG-MMSET and HA-CREB in HEK293 cells after transfection for 48 h immunoprecipitated using anti-FLAG antibody, and **b** reversely immunoprecipitated with anti-HA antibody. **c** Endogenous CREB and MMSET interaction in MM cells using MMSET antibody or **d** reversely using CREB antibody for immunoprecipitation. **e** Mapping of the interaction domain in MMSET required for CREB. **f** Interactions of HA-CREB and a series of FLAG-MMSET truncations (upper panel), and the lower panel shows expression profiles of FLAG-MMSET truncations. **g** Immunofluorescence staining of CREB and MMSET in MM.1S cells to show the subcellular co-localization. **h** Degradation of CREB in MM cells with MMSET knockdown under normoxia or hypoxia conditions. **i** Protective effects of the full length MMSET, or a SET domain depletion truncation, or a Y1179A mutant for loss of the methyltransferase function in the SET domain on CREB protein in HEK293T cells. **j** Degradation of CREB in MM cells infected with lentivirus-carrying vectors expressing full-length MMSET (FL), SET domain depletion truncation, or a Y1179A mutant for loss of the methyltransferase function in the SET domain for 72 h. Representative images are from at least *n* = 3 independent experiments for all Western blot and Co-IP assays.

Given that CREB and MMSET exert positive effects on DKK1 expression, we examined whether CREB and MMSET cooperated. We ectopically expressed HA-CREB and FLAG-MMSET in HEK293 cells and performed immunoprecipitation. We found that immunoprecipitation using an anti-FLAG antibody successfully pulled down HA-CREB (Fig. 4a) and vice versa using an anti-HA antibody (Fig. 4b), suggesting a physical association between MMSET and CREB. Notably, immunoprecipitation of myeloma cell extracts with anti-CREB or anti-MMSET antibodies also enriched MMSET or CREB proteins, respectively, providing further evidence that these two endogenous proteins form a complex in MM cells (Fig. 4c, d). We investigated the PWWP, PHD, and SET domains of MMSET, which are responsible for its interactions with other proteins (Fig. 4e). Our results indicated that only full-length MMSET and truncations without deletion of the PHD1 domain were capable of associating with CREB (Fig. 4f), suggesting that the PHD1 domain is indispensable for the interaction of MMSET with CREB. Immunofluorescence staining also showed the nuclear co-localization of CREB and MMSET after exposure to hypoxia (Fig. 4g). Intriguingly, our results also showed that when MMSET was knocked down by a lentivirus-expressing shRNA (Supplementary Fig. 3A), the extended prolonged degradation of CREB protein under hypoxia was clearly attenuated (Fig. 4h), and the extended half-life of CREB was reduced significantly (Supplementary Fig. 3B).

To define whether the protective effect of MMSET on CREB is methylation-dependent, the exogenetic CREB-expressing vector was co-expressed with vectors expressing the full-length MMSET, a SET domain depletion truncation, or a Y1179A mutant for loss of the methyltransferase function in the SET domain in HEK293T cells, and we found that only the full-length MMSET, and not methyltransferase inactive mutations, had a protective effect on CREB protein (Fig. 4i). Similarly, when the vectors expressing full-length MMSET or methyltransferase inactive mutations were delivered into MM cells using lentivirus, we found that only the full-length MMSET prolonged the half-life of CREB protein (Fig. 4j; and Supplementary Fig. 3C). Collectively, our results indicated a physical interaction between CREB and MMSET and that the methyltransferase activity of MMSET is essential to CREB stability.

### MMSET modifies histone methylation on the DKK1 promoter

**Fig. 5:**
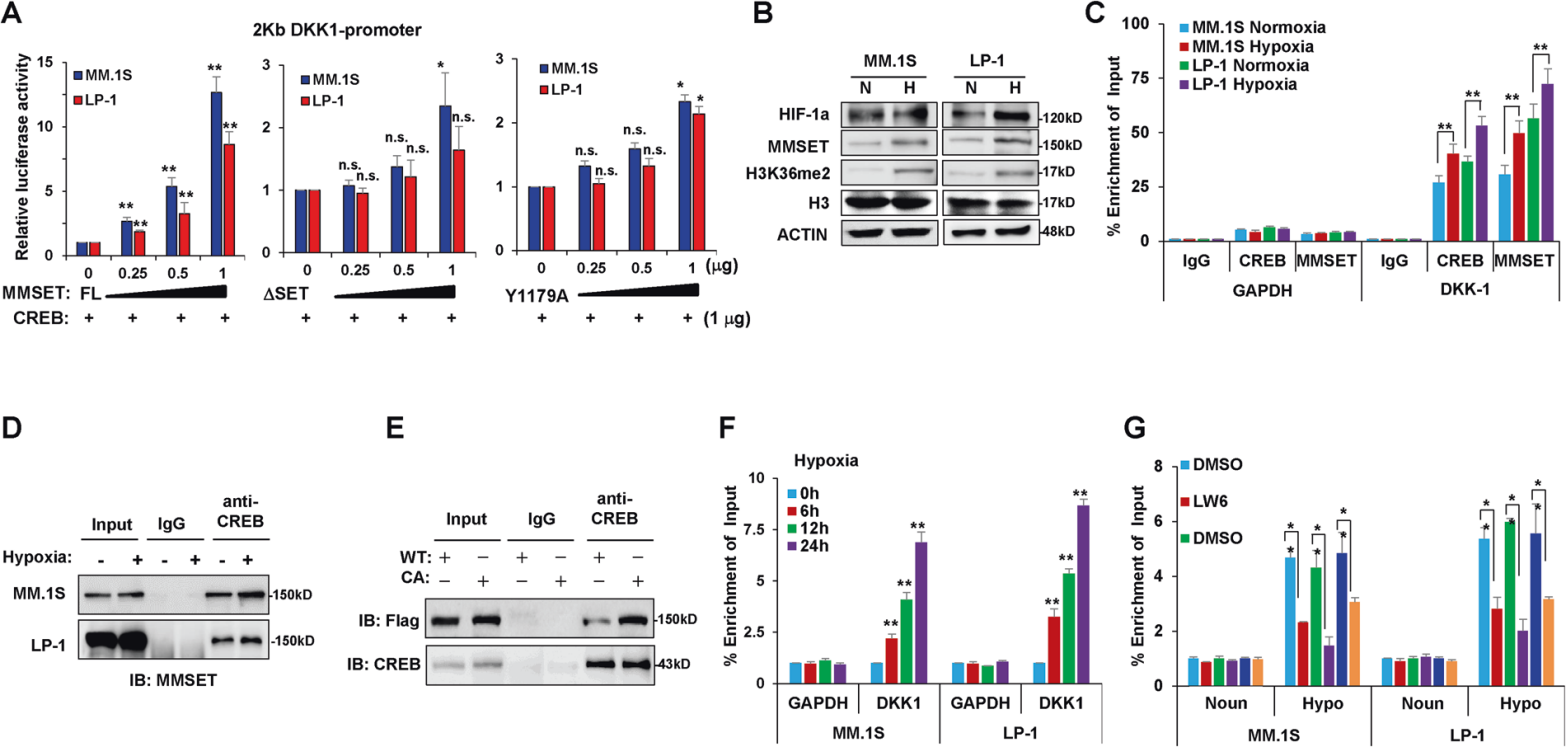
MMSET modifies the histone methylation on *DKK1* gene promoter. **a** Luciferase assay of *DKK1*-luc reporter in MM cells electroporated with plasmids encoding CREB and MMSET full length (FL), depletion of SET domain (ΔSET), or tyrosine to alanine mutation at 1179 amino acid site governing MMSET methyltransferase activity (Y1179A). **b** The MMSET and histone H3K36me2 levels in MM cells under normoxia or hypoxia conditions. **c** The enrichment of CREB and MMSET on the promoter of *DKK1* in MM cells in hypoxia condition by ChIP-qPCR, and *GAPDH* gene was used as negative control. **P < 0.001 by Student’s *t*-test for *n* = 3 independent experiments. Co-immunoprecipitation shows **d** interaction between MMSET and CREB in MM cells under hypoxia condition for 24 h, and (**e**) in HEK239T cells ectopically expressed MMSET-3flag and wild type CREB (WT) or a constitutively active CREB mutant at S133D (CA). **f** Levels of H3K36me2 on the promoter of *DKK1* gene in MM cells under hypoxia for up to 24 h by ChIP-qPCR, and **g** shows H3K36me2 levels on the promoter of *DKK1* gene in MM cells exposure to hypoxia in the presence or absence of LW6 (10 μM), SB203580 (0.5 μM), or KG-501 (10 μM) for 24 h. *GAPDH* gene was used as negative control for both experiments. **P < 0.001 by Student’s *t*-test for *n* = 3 independent experiments.

To determine whether the interaction of MMSET with CREB affects the transcriptional activity of the *DKK1* promoter, we performed reporter gene assays in MM cells. With CREB present, while the ectopic expression of full-length MMSET protein resulted in a greater than eightfold activation of the *DKK1*-luciferase reporter in MM.1S and LP-1 cells, when the SET domain was deleted or loss of function mutated, the activation of the *DKK1*-luciferase reporter was very limited (Fig. 5a). Because MMSET is a histone methyltransferase, we analyzed histone modifications after exposure to hypoxia. As MMSET was increased under hypoxic conditions, it was also evident that hypoxia increased the global levels of H3K36 methylation in myeloma cells (Fig. 5b). We further confirmed the functional association of CREB and MMSET on the *DKK1* promoter using a chromatin immunoprecipitation (ChIP) assay. Indeed, hypoxia triggered the enrichment of CREB and MMSET on the *DKK1* promoter (Fig. 5c), based on the fact that hypoxia also enhanced the interaction between CREB and MMSET (Fig. 5d). In addition, Co-IP assay using a consecutively activation mutation of CREB (S133D), which mimics hypoxia-induced phosphorylation of CREB, also indicated that the CA CREB had stronger combination with MMSET than the wild-type CREB (Fig. 5e). As a consequence, the enrichment of the *DKK1* promoter by H3K36me2 was also gradually increased under hypoxia (Fig. 5f), whereas treatment with the HIF-1α inhibitor LW6, p38 inhibitor SB203580, or CREB inhibitor KG-501 abolished the hypoxia-induced increase of H3K36me2 levels (Fig. 5g). These data indicated that CREB recruited MMSET to modify histone methylation on the *DKK1* promoter to reinforce transcription.

### Targeting CREB in myeloma cells attenuates hypoxia-induced DKK1 expression and bone disruption

**Fig. 6:**
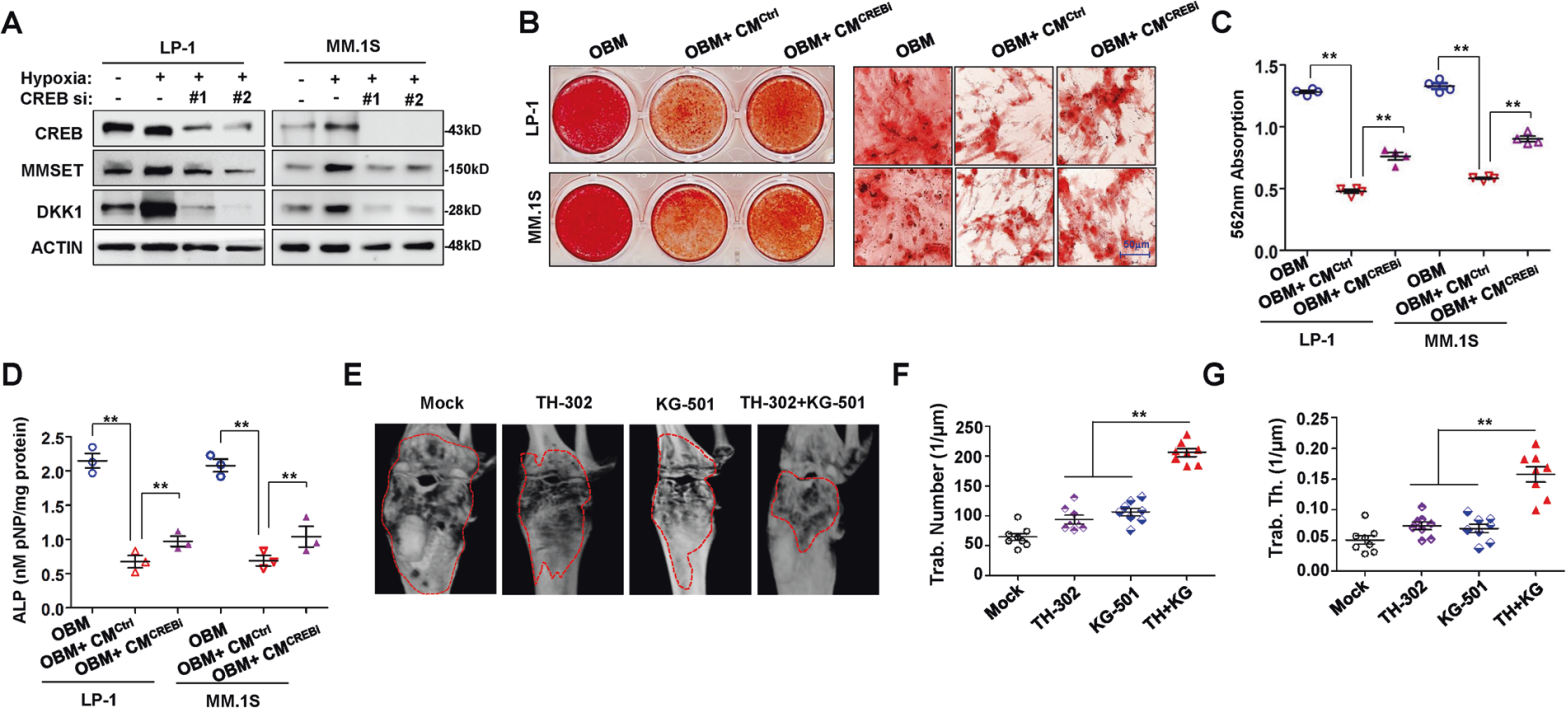
Inhibition of CREB alleviates suppression of osteoblastogenesis by myeloma cells. **a** Western blot shows MMSET and DKK1 protein levels in MM cells with CREB knockdown under hypoxia condition. **b** Alizarin Red S staining shows the osteogenesis from healthy MSCs in osteoblast medium (OBM) with addition of culture media from MM cells with *CREB* gene silenced (CM^CREBi^) or non-target control (CM^Ctrl^), and right panel showed the 200× magnification. **c** Quantification of mineralization of Alizarin Red staining in (**b**), and **d** ALP assay shows the osteoblast activity in the above groups. **P < 0.001 by Student’s *t*-test for *n* = 3 independent experiments. **e** MicroCT scan shows the bone lesion in femur bearing bortezomib-resistant MM.1S cells in NSG mice (*n* = 8) treated with 20 mg/kg TH-302 or 10 mg/kg KG-501 or combination for 1 month. Measurement of (**f**) number of bone trabecula and (**g**) the trabecular thickness in the metaphyseal region of the mice femur in the above groups. **P < 0.001 by Student’s *t*-test for *n* = 8 mice.

To determine whether targeting CREB relieves hypoxia-stimulated myeloma cell suppression of osteoblast (OB) differentiation in vitro, we knocked down CREB in myeloma cells using a lentivirus expressing shRNA, and we ensured that DKK1 expression was largely eliminated (Fig. 6a), but the MMSET protein stability was not obviously altered (Supplementary Fig. 4A). We then collected the supernatant from cell cultures of hypoxia-treated myeloma cells with different secreted DKK1 levels (Supplementary Fig. 4B), and we added it to OB medium for culture of bone marrow MSCs. Culturing MSCs with the supernatant from myeloma cells carrying non-target control shRNA (CM^Ctrl^) clearly inhibited OB activity, but the culture supernatant from myeloma cells carrying CREB shRNA (CM^CREBi^) reduced the suppression of mature osteoblasts (Fig. 6b), as evidenced by measuring Alizarin Red-S staining intensity (Fig. 6c) and the activity of alkaline phosphatase (ALP) (Fig. 6d). Meanwhile, NSG/SCID mice bearing bortezomib-resistant MM.1S cells in femur bone marrow were treated with either the hypoxia-activated prodrug TH-302 or the CREB inhibitor KG-501 or their combination. Our results indicated the remarkable remission of bone lesions in the combination group compared with the TH-302 or KG-501 alone groups (Fig. 6e). Quantitative analysis of bone structure revealed the significant recovery of trabecula disruption in the combination group, with significantly higher numbers of trabeculae (Fig. 6f) and trabecula thickness (Fig. 6g), confirming the observations with micro-computed tomography (microCT). Altogether, these data suggested that pharmacological targeting of hypoxia and CREB together could abrogate chemoresistance-induced bone lesions in vivo.

## Discussion

This study aimed to identify potential mechanisms responsible for *DKK1* overexpression in pathophysiological hypoxic conditions, such as in chemotherapy. The results showed that *DKK1* was regulated by hypoxia and epigenetic modifications. As a response to hypoxic conditions, the p38 cascade activated CREB and drove its nuclear translocation, which resulted in the cooperation of CREB with the histone methyltransferase MMSET to enhance H3K36me2 levels on the *DKK1* promoter and increase its transcriptional activation. On the basis of this theory, our study also provided in vitro and in vivo evidence for targeting hypoxia and CREB to alleviate myeloma-associated bone disease. Thus, our results provide a basis for novel therapeutic strategies to overcome chemoresistance and osteolytic lesions in patients with myeloma.

Increased DKK1 expression is a hallmark of MM, while DKK1 is almost undetectable in plasma cells from control subjects [7]. A previous study revealed that the induction of DKK1 expression by hypoxic stimulation contributes to the intense adaptation of glial tumor cells to environmental alterations [23]. It is well established that the bone marrow microenvironment of myeloma is actually quite hypoxic, a condition required for the survival and proliferation of myeloma cells, especially during the initial stage of the disease [24]. Notably, it has been shown that HIF-1α is constitutively expressed in myeloma, even under normoxic conditions. It seems that the aberrant activation of HIF-1α is a malignant feature of myeloma, and this further supports the growth of myeloma cells. Moreover, hypoxic myeloma cells usually exhibit stronger resistance to conventional chemotherapeutic agents, conferring on them highly aggressive and metastatic traits [19, 25]. Thus, harnessing the hypoxic responses of myeloma cells is a promising therapeutic strategy for MM [26, 27]. A study suggested that HIF-1α inhibition in myeloma cells can restrain tumor growth in vivo, which is accompanied by reduced angiogenesis and bone destruction [28].

In this study, we found that hypoxia elicited the rapid and persistent phosphorylation of CREB at Ser133, an essential event for CREB-mediated transcriptional activation. We also showed that p38 kinase was responsible for CREB phosphorylation and drove the nuclear shuttling of CREB; thus, our results identified CREB as a critical transcription factor linking p38 activation and *DKK1* expression under hypoxic conditions, which has not been reported previously. CREB has been implicated in the chemoresistance and tumorigenesis of MM [29]. This study provides further evidence for the important role of CREB in myeloma bone disease. Attenuating CREB activation by p38 or CREB inhibitors resulted in the dramatic repression of *DKK1* expression. Combined treatment with a CREB inhibitor and bortezomib greatly repressed the growth of myeloma cells engrafted in SCID-hu mice and significantly alleviated bone destruction.

Our investigation further revealed that epigenetic modifications also contribute to the induction of *DKK1* expression in response to hypoxia. Hypermethylation of the CpG islands of the *DKK1* promoter contributes to the absence of *DKK1* expression in various tumors, including leukemia, and the DNA demethylating agent 5-aza-2-deoxycytidine reduces promoter methylation and restores *DKK1* expression [30]. Meanwhile, genistein has been reported to induce DKK1 expression by the acetylation of histone H3 in the *DKK1* promoter region in colorectal cancer [31]. The significance of MMSET in tumorigenicity was first highlighted by the identification of the t(4;14) chromosomal translocation in approximately 15% of patients with myeloma, which links the *MMSET* gene to the immunoglobulin heavy-chain promoter, causing a dramatic increase in *MMSET* expression [32]. In myeloma cells, MMSET overexpression correlates with an increase in dimethylation at H3K36me2 and a decrease in H3K27me3 across the genome, leading to a looser chromatin structure [33]. In this study, we found that hypoxia promoted MMSET expression despite the presence of the t(4;14) cytogenetic abnormality and increased H3K36me2 levels at the *DKK1* promoter in myeloma cells. We found that *MMSET* inhibition directly decreased *DKK1* expression, and HIF-1α silencing effectively decreased *MMSET* expression, indicating that *MMSET* is a downstream gene of HIF-1α. Moreover, MMSET is a strong co-activator of NF-κB, and their cooperation activates the expression of downstream genes, including IL-6, IL-8, VEGFA, cyclin D, Bcl-2, and survivin, to protect myeloma cells from chemotherapy-induced apoptosis [34]. In this study, we provided evidence that CREB can recruit MMSET, which could protect CREB protein against degradation and modify the heterochromatin status of the *DKK1* promoter to facilitate gene transcription. Although our study suggested that hypoxia induced stronger upregulation of MMSET in MM cells, whether this regulation occurs only on the translocated allele or on non-translocated allele as well needs to be further clarified. Nevertheless, our study provides new insights into the orchestral cooperation of genetic and epigenetic regulation on *DKK1* expression under hypoxic or chemoresistance conditions.

## Materials and methods

### Patient samples

The International Myeloma Working Group criteria were used to diagnose patients with MM [35], and the key exclusion criteria were adopted accordingly [36]. Bone marrow specimens were taken as aliquots from patients with myeloma for routine examination, and plasma was taken for ELISA assay, both according to our previous procedures [37].

### Cell lines

Cell lines and cultures have been described in our previous report [37]. For hypoxia induction, MM cells were placed in a hypoxic chamber (Coy Laboratory Products, Grass Lake, MI, USA) and gassed with 95% N_2_/5% CO_2_ at 37 °C for different durations. Cell lines were authenticated by short tandem repeat DNA profiling (Shanghai Biowing Applied Biotechnology), and mycoplasma-free condition was secured before further experiments.

### Real-time PCR and western blot assays

Normoxia and hypoxia myeloma cells RNA was isolated using Trizol (Ambion, Carlsbad, CA, USA) according to the manufacturer’s instructions. Details of qPCR and western blot procedures can be found in our previous report [37]. The primers used in qPCR and antibodies used in this study are listed in Supplementary Table 1.

### Immunohistochemistry and immunofluorescence staining

IHC staining was carried out as in our previous study [37]. Detection of DKK1, CREB, and MMSET was achieved by using the DAKO EnVision^+^ System (Agilent, Carpinteria, CA, USA). For fluorescence staining, MM cells were fixed with 4% formaldehyde and permeabilized with Triton X-100. After blocking with BSA, cells were incubated with anti-CREB or anti-MMSET antibody at 4 °C overnight, followed by incubation with Alexa 594- or Alexa 488-conjugated secondary antibodies for 30 min at room temperature and nucleus counterstaining with DAPI. Imaging was performed using a fluorescence microscope (model IX71; Olympus, Tokyo, Japan).

### Enzyme-linked immunosorbent assay (ELISA)

Supernatants from 24-h cell cultures or bone marrow plasma from patients with MM were collected, and the DKK1 levels were determined with a commercially available ELISA kit (R&D Systems, Minneapolis, MN, USA) according to the manufacturer’s procedures. Each sample was run in triplicate, and the results were obtained for three independent experiments.

### Infection, transfection, and luciferase assay

Lentivirus packaging and infection were performed as previously reported [37]. Briefly, a 50 μl viral concentration and 8 μg/ml polybrene were added to 1 × 10^6^ myeloma cells in 1 ml media for 12 h; the medium was changed, and cells were cultured for another 48 h until further management. For transient transfections of plasmids, the Neon transfection system (Invitrogen) was used. Briefly, 1 × 10^6^ myeloma cells were mixed with 10 μg of plasmids, and the electroporation was performed under the condition of 1 600 V, 20 ms, and 1 pulse. For the luciferase assay, MM cells were transfected with DKK1-luc and pRL-TK Renilla plasmids together with plasmids encoding CREB and/or MMSET. The cell lysate was used to detect luciferase activity in a Dual-Luciferase Reporter assay system (Promega, Madison, WI, USA) according to the manufacturer’s protocol.

### In vitro osteoblast formation and function assays

Mature osteoblasts were generated from MSCs in a standard 7- to 14-day culture with OB medium as described previously [38]. To examine the effects of DKK1 secreted from myeloma cells on OB differentiation, MSCs were cultured in OB medium with or without myeloma cell cultures (conditioned media) at a ratio of 1:1. The maturity of the osteoblasts was determined by measuring ALP activity and Alizarin Red staining as previously reported [39]. The images were scanned and captured for each well by the Olympus IX71 fluorescence microscope (Olympus, Tokyo, Japan).

### Immunoprecipitation and chromatin immunoprecipitation (ChIP) assays

Immunoprecipitation was performed as previously reported [37]. HEK293T cells were transfected with HA-CREB vectors or a series of FLAG-MMSET fragments. For ChIP-qPCR assay, chromatin samples were immunoprecipitated with antibodies against CREB, MMSET, H3K36me2, or control IgG at 4 °C overnight, and immunoprecipitates or total chromatin input were isolated and analyzed by real-time PCR with primers specific for the promoter regions of *DKK1* gene (Supplementary Table 1).

### NOD-*scid* IL2Rg^null^ (NSG)-bearing mouse bone lesion model

Female NSG mice at 8 weeks of age were randomized blindly into four groups to establish the bone lesion model. Bortezomib-resistant MM.1S cells (5 × 10^5^/mouse) were injected into the femurs of NSG mice. After 3 weeks, mice were intraperitoneally treated with CREB inhibitor KG-501 (10 mg/kg) (*n* = 8) or TH-302 (20 mg/kg) (*n* = 8), solo or together, every 2 days for another 2 weeks. After 24 days of treatment, mouse femurs were subjected to microCT scans with a SkyScan 1276 microtomograph. Trabecular bone analysis was performed to calculate the trabecular number (Tb.N, in µm^−1^) and trabecular thickness (Tb.Th, in µm).

### Statistical analysis

The effective sample size was determined using SPSS 13.0 software according to a previous report [40], in which the power of the study was 80%, and the level for type I error was 5% (*P* < 0.05). Data are shown as mean ± SD for three independent experiments, with each sample triplicated. Differences between groups were determined using a paired two-tailed Student’s *t*-test, and one-way ANOVA plus a Bonferroni post hoc test were used for all of the experiments. A Pearson correlation test was used to determine the correlations between gene expressions, and survival analysis was performed using GraphPad Prism 5.0. A *P* value less than 0.05 was considered to be statistically significant.

Additional detailed materials and methods are listed in the Supplementary methods.

## Conclusion

In summary, this study revealed that the cooperation of the p38-CREB cascade with MMSET promotes DKK1 expression in response to hypoxia in myeloma cells. Regulating DKK1 expression by targeting hypoxia and CREB together may have therapeutic significance in the management of myeloma patients with chemoresistance and lytic bone disease.

## Supplementary information

Supplementary Figure 1–4

Supplementary methods

Supplementary Table 1

Supplementary Table 2
