# Supplementary material for: Hypoxia-induced CREB cooperates MMSET to modify chromatin and promote DKK1 expression in multiple myeloma
Source: Oncogene. 2021 Jan 8;40(7):1231–41. doi: 10.1038/s41388-020-01590-8 (PMC7892339; doi:10.1038/s41388-020-01590-8)
Supplement: Supplementary file 1 — Supplementary Figure 1–4 [file 41388_2020_1590_MOESM1_ESM.docx]

**Supplementary Figures**


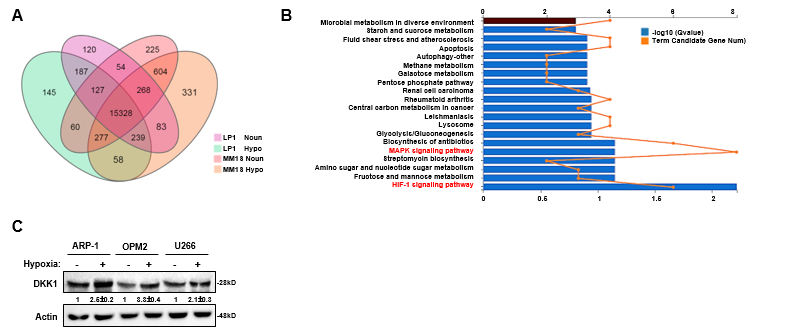
 **S Figure 1. Transcriptome profiling of myeloma cells in response to hypoxia.** (A) Overlap of differentially expressed genes of MM.1S and LP-1 cells under normoxia (Noun) and hypoxia (Hypo) conditions. (*P* value <0.05 and abs (log 2FC) >1, n=3). (B) KEGG analysis showed the mostly affected pathways under hypoxia stimulation. (C) Representative Western blotting images shows DKK1 expression in MM cell lines with different cytogenetic background under hypoxia stimulation for 24hr, ARP-1with t(14;16), OPM2 with t(4;14), and U266 with t(11;14). Number under the DKK1 blots is the mean±SD of three independent experiments using densitometric analysis in an Image J software.


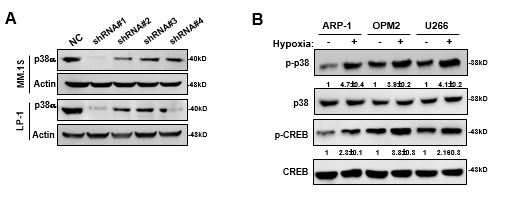


**S Figure 2. Activation of p38 and CREB in myeloma cells with different cytogenetic background.** (A) Western blotting images shows the knockdown efficacy of p38α (*MAKP14* gene) using lentivirus carrying shRNAs in MM.1S and LP-1 cells for 72 hr. (B) Representative Western blotting images shows phosphorylation levels of p38 (p-p38) and CREB (p-CREB) in MM cell lines with different cytogenetic background under hypoxia stimulation for 24hr, ARP-1with t(14;16), OPM2 with t(4;14), and U266 with t(11;14). Number under the DKK1 blots is the mean±SD of three independent experiments using densitometric analysis in a Image J software.


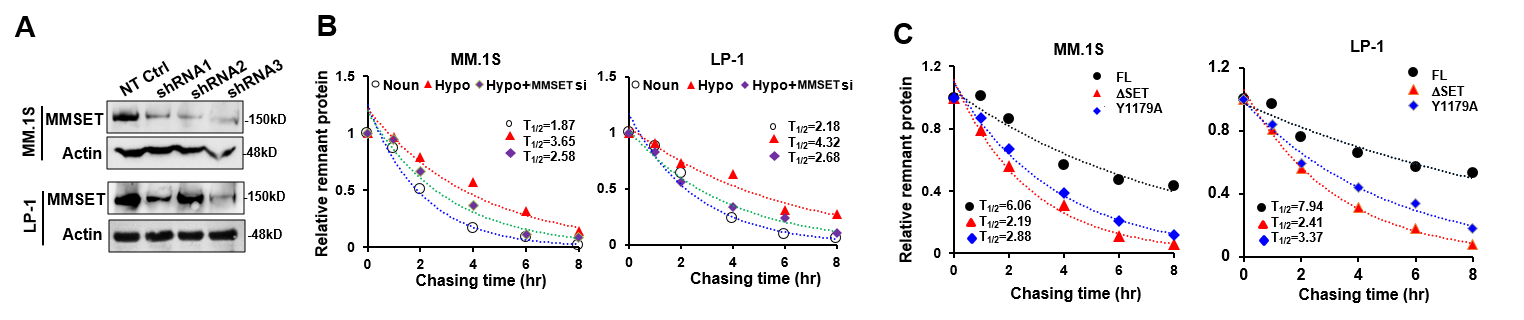


**S Figure 3. MMSET physically interacts with and protects CREB in myeloma cells.** (A) Efficacy of MMSET knockdown by lentivirus expressing short hairpin RNAs (shRNA1, shRNA2, shRNA3) in MM.1S and LP-1 cells. Non-target control (NT Ctrl) shRNA was used as control. (B) Calculated half-life (T1/2) of CREB protein in MM.1S and LP-1 cells under normoxia (Noun) or hypoxia (Hypo) conditions with or without MMSET knockdown (MMSET^KD^). (C) Calculated half-life (T1/2) of CREB protein in MM.1S and LP-1 cells with overexpression of vectors expressing full length MMSET (FL), SET domain deleted truncation, or a Y1179A mutant for loss of the methyltransferase function in the SET domain.


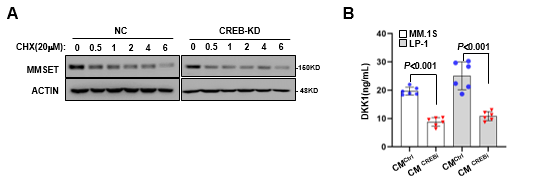


**S Figure 4. MMSET protein stability and secreted DKK1 level in culture media of MM cells.** (A) MMSET protein stability in LP-1 cells. LP-1 cells were infected with lentivirus carrying shRNA targeting CREB (CREB-KD) or non-target control (NC) for 72hr, and then cells were treated 20μM of cycloheximide (CHX) for up to 6 hr. (B) ELISA assay detecting secreted DKK1 level in culture media of MM.1S and LP-1 cells infected with lentivirus carrying shRNA targeting CREB (CREBi) or non-target control (Ctrl) for 72 hr. Data were from two independent experiments run in triplicate (n=6). Difference between groups were analyzed by student *t*-test.
