# Supplementary material for: Hypoxia-induced CREB cooperates MMSET to modify chromatin and promote DKK1 expression in multiple myeloma
Source: Oncogene. 2021 Jan 8;40(7):1231–41. doi: 10.1038/s41388-020-01590-8 (PMC7892339; doi:10.1038/s41388-020-01590-8)
Supplement: Supplementary file 2 — Supplementary methods [file 41388_2020_1590_MOESM2_ESM.docx]

**Methods**

**Cell lines**

Cell lines and cultures have been decbribed in our previously reported[[17](#_ENREF_17)]. Myeloma cell line LP-1 was purchased from ATCC (American Type Culture Collection, Manassas, VA, USA), MM.1S and U266 were from the National Infrastructure of Cell Line Resource (Beijing, China), OPM2 was kindly provided by Dr. Jinsong Hu at Xi’an Jiaotong University, and ARP1 was a gift from Dr. Yong Lu at the Wake Forest University Health Care. MM cells were cultured in RPMI-1640 media supplemented with 15% of fetal bovine serum, 100 U/mL of penicillin, 100 mg/mL of streptomycin, and 2 mM L-glutamine (Gibco, Life Technologies, Carlsbad, CA, USA). The HEK293T cells was cultured in DMEM-high glucose media with 10% fetal bovine serum, 100 U/mL of penicillin, 100 mg/mL of streptomycin, and 2 mM L-glutamine. These cells were all cultured at 37℃ in a humidified incubator with 5% CO_2_ (Gibco, Life Technologies, Carlsbad, CA, USA). For hypoxia induction, MM cells were placed in a hypoxic chamber (Coylab, Grass Lake, MI) and gassed with 95% N_2_/5% CO_2_ at 37°C for different times. Cell lines were authenticated by short tandem repeat DNA profiling (STR) (Shanghai Biowing Applied Biolotechnology), and mycoplasma free was secured before further experiments.

**Establishment of Bortezomib (BTZ)-resistant myeloma cells**

To develop bortezomib-resistant (BR) myeloma cells, parental drug-naive cells were imitated by 0.5 nM of BTZ and enhanced by doubled dosage very one month up to six months totally. Acquire of BR-resistant phenotype were monitored and confirmed by calculating the IC_50_ of BTZ using MTS assay. Cells with IC_50_ over 10 times were kept for further experiments.

**Western blotting**

Details of Western blot procedure can be found in our previous report[[17](#_ENREF_17)]. Protein lysates were prepared in RIPA-buffer (50 mM Tris-Hcl pH 7.5, 150 mM NaCl, 10 mM EDTA, 0.5% sodium deoxycholate, 1% NP-40, 1 mM sodium ovanadate, 10 μg/mL aprotinin, 1 mM phenylmethanesulfonyl fluoride, and 10 μg/mL leupeptin) supplemented with complete protease inhibitors (Roche, Indianapolis, IN, USA). The protein concentration was determined using the BCA protein assay kit (ThermoFisher Scientific, Carlsbad, CA, USA). Cell lysate (50 μg) was separated by electrophoresis on SDS-PAGE gel and transferred to nitrocellulose membranes (Pall Corporation, Washington, NY, USA). Membranes were blocked with 5% non‑fat milk for 1 hr at room temperature and probed overnight at 4°C with specific antibodies. Antibodies used in this study were listed in the **supplementary resources (S Table 1)**. Membranes were washed three times in PBST the next day, then incubated with horseradish peroxidase-conjugated secondary antibodies for 1 hr at room temperature, washed three times with PBST and finally bands were visualized using an enhanced chemiluminescence system (Millipore, Los Angeles, CA USA). The representative western blot images for at least three independent experiments shown in the figures have been cropped and auto contrasted. The antibodies used in this study were listed in the **supplementary resources (S Table 1).** Densitometric analysis of blots were using Image J software (NIH, USA).

**Immunohistochemistry**

IHC staining were carried out as previous study[[17](#_ENREF_17)]. Detection of DKK1, CREB, and MMSET were achieved by using the DAKO EnVision^+^ System (Angilent, Carpinteria, CA, USA). Deparaffinize myeloma tissue array with normal bone marrow tissue slides in xylene for 2 times, 15 min each. Transfer slides to 100% alcohol, for 2 times, 5 min each, and then transfer once through 95%, 70% and 50% alcohols sequentially for 5 min each. Block endogenous peroxidase activity by incubating sections in 3% H_2_O_2_ solution at room temperature for 10 min to block endogenous peroxidase activity. Rinse with PBS twice, 5 min each. Pour 10 mM citrate buffer pH 6.0 into the staining container and incubate it at 98°C for 20 min. Remove the staining container to room temperature and allow the slides to cool for 40 min. Rinse slides with PBS for 2 times, 5 min each. Add blocking buffer onto the sections of the slides and incubate in a humidified chamber at room temperature for 1 hr. Drain off the blocking buffer from the slides. Apply appropriately diluted primary antibody to the sections on the slides and incubate in a humidified chamber at 4°C overnight. Wash the slides with PBS for 3 times, 5 min each. Apply appropriately diluted biotinylated secondary antibody to the sections on the slides and incubate in a humidified chamber at room temperature for 1 hr. Wash slides with PBS for 3 times, 5 min each. Apply DAB substrate solution (Dako, K5361) (freshly made just before use) to the sections on the slides to reveal the color of antibody staining. Allow the color development for less than 10 min until the desired color intensity is reached. Wash slides with PBS. Counterstain slides by immersing sides in Hematoxylin for 1-2 min. Rinse the slides in running tap water for 10 min. Dehydrate the tissue slides through 5 times of alcohol (50%, 70%, 95%, 100% and 100%), 1 min each. Clear the tissue slides in 2 times of xylene and coverslip using mounting solution. The mounted slides can be ready for photo capture or stored at room temperature permanently.

**Immunofluorescence staining**

MM cells were fixed with 4% formaldehyde for 10 minutes , then samples were treated in 0.5% (V/V) Triton X-100 for 15 min and blocked with 5% BSA for 30 min at 37°C. After incubated with anti-CREB or anti-MMSET antibody overnight at 4°C, followed by incubation with Alexa594- or Alexa488-conjugated secondary antibodies (1:2000) for 60 min at room temperature and nucleus counterstaining with DAPI. Imaging was obtained by the Olympus FV1000 IX81-SIM Confocal Microscope (Olympus, Tokyo, Japan).

**Enzyme linked immunosorbent assay (ELISA)**

To detect the DKK1 levels, specimen were measured in supernatants from cell cultures or in bone marrow plasma from MM patients with commercially available ELISA kit (R&D Systems, Minneapolis, MN, USA). Briefly, bone marrow plasma were diluted with sample dilution buffer and added into the plate with primary antibody for incubation for 2 hr at room temperature. Afterward, secondary antibody were prepared accordingly and added into samples for 2 hr at room temperature. Then, the substrate was added to develop the signal for detection. Determine the optical density (OD value) of each well at once with a micro-plate reader set to 450 nm. Each sample were triplicated and the results were represented for two independent experiments.

**Transfection, virus package and infection**

Transfection, lentivirus packaging and infection were performed as previously reported[[17](#_ENREF_17)]. Transient transfections to HEK293T cells were performed using polyethyleneimine (PEI) (Polysciences, Warrington, PA, USA) in the OPTI-MEM medium (Life Technologies, Carlsbad, CA, USA) with a ratio of 1:4 to 1:6 of DNA:PEI. Transient transfections to MM cells were performed using Neon electroporation Transfection System (Invitrogen of Life Technologies, Carlsbad, CA, USA) according to the manufacturer’s instructions. Briefly, 2×10^6^ cells were mixed with total 10 μg plasmids in 150 μL resuspend buffer, and electroporated under the condition: voltage=1600V, width=20ms, pulses=1. Cells were changed to complete media 6 hr after transfection, and collected 48 h post transfection for further designed experiments.

Viral particles were produced by HEK293T cells in a 10 cm dish transfected with 4 μg PMD2G and 6 μg PSPAX2 packaging plasmids (Addgene, Watertown, MA, USA) , together with 8 μg lentiviral expressing vectors encoding target genes, including pCMV-C-HA-CREB, pLV-C-FLAG-MMSET or pLKO.1 vector encoding shRNA targeting *MMSET*. Supernatant carrying the viral particles was harvested 35 hr and 60 hr after transfection and concentrated to 100× volume by Poly (ethylene glycol) 8,000 (Sigma-Aldrich, St. Louis, MS, USA).

For viral infection, 1×10^6^ myeloma cells were seeded in 1 mL new complete media for 6 hr and then added 50 μL viral concentration and 8 μg/mL polybrene, and cells were spin at 1800 rpm for 45 minutes at 20°C. 12 hr after spinfection, the medium was changed and cells were cultured for another 48 hr until further management.

**Luciferase assay**

For luciferase assay, 0.8 μg of total DNA including 0.2 μg DKK1-luc reporter vector and 1 ng pRL-TK Renila plasmid as internal control together with plasmids encoding CREB and /or MMSET, or empty vectors were mixed with 1×10^6^ myeloma cells in suspending buffer to the final volume of 120 µL and then electroporated using a Neon Transfection System (ThermoFisher Scientific, Waltham, MA, USA). 48 hr after transfection, cell lysate was used to detect luciferase activity in a Dual-Luciferase Reporter assay system (Promega, Madison, WI, USA) according to the manufacturers’ protocols, and signal captured in the SpectraMax M5 multi-detection microplate reader (Molecular Devices Corporation, Sunnyvale, CA, USA).

**In vitro osteoblast formation and function assays**

Mature osteoblasts were generated from MSCs in a standard 14-day culture with osteoblast medium (OB medium) as described previously[[18](#_ENREF_18)]. MSCs were cultured in a 6-well plate with complete medium. After reaching 80% confluence, the medium was changed to osteogenic differentiation medium in presence or absence of 1 μmol/L melatonin for 14 days with a medium change every 3 days. The osteogenic differentiation medium was composed of high-glucose DMEM, penicillin (100 U/mL), streptomycin (100 µg/mL), dexamethasone (0.1 μmol/L), 10% FBS, ascorbic acid (50 μg/mL), and β-glycerol phosphate (10 mmol/L). To examine the effects of secreted DKK1 from myeloma cells on osteoblast differentiation, MSCs were cultured in OB medium with or without myeloma cell cultures (conditioned media, CM) at a ratio of 1:1. The maturity of the osteoblasts was determined by measuring alkaline phosphatase (ALP) activity and Alizarin Red staining as previously reported[[19](#_ENREF_19)]. The images were scanned and captured for each well by the Olympus FV1000 IX81-SIM Confocal Microscope (Olympus, Tokyo, Japan).

**Immunoprecipitation**

Immunoprecipitation was performed as previously reported[[17](#_ENREF_17)]. HEK293 cells were transfected with HA-CREB vectors or a series of FLAG-MMSET fragments. Cells were harvested and lysed by NP-40 lysis buffer (50 mM Tris-Hcl pH 7.4, 150 mM NaCl) supplemented with complete protease inhibitors (Roche, Indianapolis, IN, USA) on ice for 30 min. Cell lysate was centrifuged for 20 min at 12,000g at 4°C. Co-IP for exogenous expressed proteins, supernatant was incubated with anti-FLAG M2 Affinity Gel (Sigma-Aldrich, St. Louis, MO, USA) or EZview™ Red Anti-HA Affinity Gel (Millipore, Los Angeles, CA USA), for endogenous assays myeloma cells, the supernatant was incubated with anti-CREB antibody (2 μg) or anti-MMSET antibody (2 μg) at 4°C overnight with protein G dynabeads (ThermoFisher Scientific, Carlsbad, CA, USA). The next day, the pellet was washed four times with NP-40 lysis buffer, and then subjected to Western blotting analysis using the anti-MMSET or anti-CREB antibodies, respectively.

### Chromatin immunoprecipitation assays (ChIP)

40 million cells were washed in PBS and cross-linked with 1% formaldehyde for 10 min at room temperature and then quenched by addition of glycine (125 mM final concentration) for 5 min. For Nuclei isolation, cells were resuspended in cell lysis buffer (50mM Tris pH8.0, 140 mM NaCl, 1mM EDTA, 10% glycerol, 0.5% NP-40, 0.25% Triton X-100), incubated the tube on ice for 20 min to swell. Harvested the nuclei by centrifugation at 2000g for 5 min at 4°C resuspended in 1 mL ChIP lysis buffer (1% SDS, 10mM EDTA, 50mM Tris-HCl, pH8.0) and incubated on ice for 10 min. Chromatin was fragmented to 200-500 bp using 12 cycles using the Vibra-Cell Ultrasonic Liquid Processors (SONICS, Newtown, CT, USA). For each IP, chromatin was immunoprecipitated with 2 µg of antibodies against CREB, MMSET, H3K36me2 or control IgG in IP dilution buffer (1% Triton X-100, 2mM EDTA, 150mM NaCl, 20mM Tris-HCl, pH 8.0) at 4°C overnight. Chromatin was precleared for 2 hr each with protein G agarose beads (Cell Signaling Technology，Danvers, MA, USA) before immunoprecipitation. The immunoprecipitated material was washed, once in TSE I buffer (20mM TrisHCl pH 8.0, 2mM EDTA pH8.0, 150mM NaCl, 1% Triton X-100, 0.1% SDS), once in TSE II buffer (20mM TrisHCl pH 8.0, 2mM EDTA pH8.0, 500mM NaCl, 1% Triton X-100, 0.1% SDS), once in LiCl buffer (10mM TrisHCl pH 8.0, 250mM LiCl, 1% deoxycholic acid, 1% NP40) and once in TE buffer (10mM Tris pH 8.0, 1mM EDTA pH8.0) before elution in elution buffer (100mM NaHCO3, 1% SDS). The samples were removed from beads, reversed cross-linked overnight at 65°C and DNA was isolated using QIAquick PCR Purification Kit (Germantown, MD, USA). Immunoprecipitates or total chromatin input were isolated and analyzed by real-time PCR with primers specific for the promoter regions of *DKK1* gene.
