# Supplementary material for: Hypoxia-induced CREB cooperates MMSET to modify chromatin and promote DKK1 expression in multiple myeloma
Source: Oncogene. 2021 Jan 8;40(7):1231–41. doi: 10.1038/s41388-020-01590-8 (PMC7892339; doi:10.1038/s41388-020-01590-8)
Supplement: Supplementary file 3 — Supplementary Table 1 [file 41388_2020_1590_MOESM3_ESM.docx]

**Key resources of this study**

| **Category** | **Source** | **Cat. No.** |
| --- | --- | --- |
| **Antibodies** | | |
| Anti-Histone H3 (di methyl K36) antibody-ChIP Grade | Abcam | ab9049 |
| Anti-WHSC1/NSD2 antibody [29D1] - ChIP Grade | Abcam | ab75359 |
| Anti-rabbit β-actin | Abclonal | AC006 |
| Goat Anti-Rabbit IgG-HRP | Sigma-Aldrich | A0545 |
| Rabbit Anti Mouse IgG-HRP | Sigma-Aldrich | A9044-2ML |
| Anti-Histone H3 antibody - Nuclear Loading Control and ChIP Grade | Abcam | ab1791 |
| Anti-mouse IgG | Proteintech | B900620 |
| Anti-FLAG® M2-Peroxidase | Sigma-Aldrich | A8592 |
| Anti-3FLAG peptide | Sigma-Aldrich | F4799 |
| Anti-HIF-1α (D1S7W) XP® Rabbit antibody | Cell Signaling Technology | #36169 |
| Anti-HA-Tag (C29F4) Rabbit antibody (Biotinylated) | Cell Signaling Technology | #5017 |
| Anti-p38 MAPK (D13E1) XP® Rabbit antibody | Cell Signaling Technology | #8690 |
| Anti-Phospho-p38 MAPK (Thr180/Tyr182) (D3F9) XP® Rabbit antibody | Cell Signaling Technology | #4511 |
| Anti-Akt (pan) (40D4) Mouse antibody | Cell Signaling Technology | #2920 |
| Anti-Phospho-Akt (Ser473) (D9E) XP® Rabbit antibody | Cell Signaling Technology | #4060 |
| Anti-DKK1 (D5V6L) Rabbit antibody | Cell Signaling Technology | #48367 |
| Anti-Lamin B1 (119D5-F1) Mouse antibody | Cell Signaling Technology | #68591 |
| Anti-CREB (48H2) Rabbit antibody | Cell Signaling Technology | #9197 |
| Anti-Phospho-CREB (Ser133) (87G3) Rabbit antibody | Cell Signaling Technology | #9198 |
| **Drugs** |  |  |
| Penicillin streptomycin mixed solution (100 ×) | Solarbio | P1400 |
| Puromycin 2HCL | SelleckChem | S7417 |
| β-glycerol phosphate | Sigma-Aldrich | 50020 |
| SB203580 | Selleckchem | S1076 |
| KG501 | Selleckchem | S8409 |
| LW6 | Selleckchem | S8441 |
| Evofosfamide (TH-302) | Selleckchem | S2757 |
| **Enzymes** |  |  |
| RNase A, DNase and protease-free | Thermo Fisher | EN0531 |
| Proteinase K Solution, ChIP grade | Thermo Fisher | 26160 |
| Benzonase Nuclease | Sigma-Aldrich | E1014-25KU |
| FastAP Thermosensitive Alkaline Phosphatase | Thermo Fisher | EF0651 |
| AgeⅠ | NewEngland Biolabs | R0580S |
| NotI-HF | NewEngland Biolabs | R3189S |
| BamHI | NewEngland Biolabs | R0136S |
| XbaI | NewEngland Biolabs | R0145S |
| EcoRI | NewEngland Biolabs | R0101S |
| KpnI-HF | NewEngland Biolabs | R3142S |
| HindIII | NewEngland Biolabs | R104S |
| XhoI | NewEngland Biolabs | R0146S |
| CutSmart Buffer | NewEngland Biolabs | 137204S |
| NEBuffer1 | NewEngland Biolabs | B7001S |
| NEBuffer2 | NewEngland Biolabs | B7002S |
| NEBuffer3 | NewEngland Biolabs | B7003O |
| NEBuffer4 | NewEngland Biolabs | B7004S |
| T4 DNA Ligase | NewEngland Biolabs | M0202S |
| 10×Buffer for T4 DNA ligase | NewEngland Biolabs | B0202S |
| Multiscribe Reverse Transcriptase | ABI | 4308228 |
| dNTP mix | ABI | 362275 |
| **Plasmids** |  |  |
| pCMV3-C-HA-CREB | Sino Biological | HG11530-CY |
| pCMV3-HA Vector | Sino Biological | CV013 |
| pCMV3-C-HA-CREB CA | Self-construction | |
| pCDH-CMV-MCS-EF1-copGFP | Addgene | 73030 |
| pLV-C-3FLAG-MMSET | Sino Biological | HG11530-CFLN |
| pITA insert | Gift from Dr. Yupeng Chen, Tianjin Medical University | |
| pcDNA-3×FALG | Gift from Dr. Michael Naksi lab, UT Health Science Center at San Antonio | |
| pLKO.1 vector | Gift from Dr. Feng Wang, Tianjin Medical University, Dept. Genetics | |
| MMSET-shRNA1 | Self-construction | |
| MMSET-shRNA2 | Self-construction | |
| MMSET-shRNA3 | Self-construction | |
| MAPK14-shRNA1 | Self-construction | |
| MAPK14-shRNA2 | Self-construction | |
| MAPK14-shRNA3 | Self-construction | |
| MAPK14-shRNA4 | Self-construction | |
| MMSET-ΔSET | Gift from Dr. June Qin, Shanghai Institutes for Biological Sciences | |
| MMSET-Y1179A |  |  |
| pGL3-Basic-hDKK1 promoter (2000bp) | Self-construction | |
| pGL3-Basic vector | Addgene | |
| **Critical Commercial Assays** |  | |
| EvaGreen 2× qPCR MasterMix | ABI | MasterMix-R |
| 5×All-In-One RT MasterMix | Abm | G490 |
| Luciferase assay Kit | Promega | E1910 |
| Pierce BCA Protein Assay Kit | Thermo SCIENTIFIC | 23225 |
| AxyPrep DNA Extraction Kit | AXYGEN | 295 AP-GX-250G |
| AxyPrep Plasmid Miniprep Kit | AXYGEN | 183 AP-MN-P-250G |
| Plasmid Maxi Kit(25) | QIAGEN | 12163 |
| EnVision G12 Doublestain System,Rabbit/Mouse(DAB+/Permanent Red) | Dako | K5361 |
| SuperSignal West Dura Extended Duration Substrate | ThermoFisher | 34580 |
| 9002 SimpleCHIP® Kit | Cell Signaling | 22188S |
| Simple CHIP® Kits-20C-Reagents | Cell Signaling | 45061S |
| ChIP-grade Protein A/G Magnetic Beads | Thermo SCIENTIFIC | 26162 |
| Lipofectamine 3000 Transfection Kit | Invitrogen | L3000-008 |
| Polybrene Infection / Transfection Reagent | Sigma-Aldrich | TR-1003 |
| NuPAGE 4-12% Bis-Tris Gel | Invitrogen | NP0335BOX |
| Poly (ethylene glycol) 8,000 | Sigma-Aldrich | TR-1003 |
| Alkaline Phosphatase Activity Colorimetric Assay Kit | BioVision | K412 |
| BCIP / NBT basic phosphatase reagent kit | Beyotime | C3206 |
| Alizarin red | Sigma-Aldrich | A5533 |
| Human Dkk-1 Quantikine ELISA Kit | R&D System | DKK100B |
| 10% neutral formalin buffer | Sigma-Aldrich | HT501128 |
| Cetyl pyridinium chloride | Sigma-Aldrich | 1104006 |
| Na-phosphate buffer | Sigma-Aldrich | 71640 |
| 37% formaldehyde | Sigma-Aldrich | 252549 |
| p-nitrophenyl phosphate solution | Sigma-Aldrich | 487663 |
| p-NPP Substrate Buffer | Sigma-Aldrich | 487664 |
| Ficoll-Paque PLUS endotoxin tested | GE Healthcare | 17-1440-02 |
| TRIzol Reagent | Ambion, Life Science | 15596018 |
| Opti-MEM®I(1×) Reduced Serum | Gibco, Life Technologies | 31985-070 |
| Opti-protein XL Marker | Abm | G266 |
| PageRuler Prestained protein Ladder | ThermoFisher Scientific | 26616 |
| 1Kb Ladder DNA Marker | Biomed | MD114 |
| 1Kb DNA Ladder | TIANCEN | MD111 |
| 100bp DNA Ladder | TRANS | BM301 |
| BM15000 DNA Marker | Biomed | MD106 |
| 1Kb Plus DNA Ladder | Solarbio | M1500 |
| PEI-Transferrinfection Kit | ThermoFisher Scientific | BMS1003 |
| **Primers sequence** |  |  |
| HumanGAPDH-F | TTGCCCTCAACGACCACTTT |  |
| HumanGAPDH-R | TGGTCCAGGGGTCTTACTCC |  |
| HumanMMSET-F | CTCCTGGGAGGAAGAAGACC |  |
| HumanMMSET-R | CACAGCTGGCACACATACTC | |
| HumanDKK1-F | CACACCAAAGGACAAGAAGGT | |
| HumanDKK1-R | CCACAGTAACAACGCTGGAA | |
| CHIP-qPCR DKK1 F | AGAGCCTATCACCCCTCGG | |
| CHIP-qPCR DKK1 R | TGGGAGGGAGACAACAAAGC | |
| hMMSETsh#1-F | CCGGCGGAAAGCCAAGTTCACCTTTCTCGAGAAAGGTGAACTTGGCTTTCCGTTTTTG | |
| hMMSETsh#1-R | AATTCAAAAACGGAAAGCCAAGTTCACCTTTCTCGAGAAAGGTGAACTTGGCTTTCCG | |
| hMMSETsh#2-F | CCGGATCTTACTTCCCGGGTGTTTACTCGAGTAAACACCCGGGAAGTAAGATTTTTTG | |
| hMMSETsh#2-R | AATTCAAAAAATCTTACTTCCCGGGTGTTTACTCGAGTAAACACCCGGGAAGTAAGAT | |
| hMMSETsh#3-F | CCGGCCCAGAAAGAGCTTGGATATTCTCGAGAATATCCAAGCTCTTTCTGGGTTTTTG | |
| hMMSETsh#3-R | AATTCAAAAACCCAGAAAGAGCTTGGATATTCTCGAGAATATCCAAGCTCTTTCTGGG | |
| hMMSETsh#4-F | CCGGCCCAGAAAGAGCTTGGATATTCTCGAGAATATCCAAGCTCTTTCTGGGTTTTT | |
| hMMSETsh#4-R | AATTCAAAAACCCAGAAAGAGCTTGGATATTCTCGAGAATATCCAAGCTCTTTCTGGG | |
| SimpleChIP™ Human GAPDH  Promoter Primers | Cell Signaling, #4471 | |
